# A method for evaluating discoverability and navigability of recommendation algorithms

**DOI:** 10.1186/s40649-017-0045-3

**Published:** 2017-10-11

**Authors:** Daniel Lamprecht, Markus Strohmaier, Denis Helic

**Affiliations:** 1 ISDS, Graz University of Technology, Inffeldgasse 16c, 8010 Graz, Austria; 2 GESIS-Leibniz Institute for the Social Sciences, Unter Sachsenhausen 6-8, 50667 Cologne, Germany; 3 University of Koblenz-Landau, Universitätsstr. 1, 56070 Koblenz, Germany

**Keywords:** Navigation, Recommender systems, Decentralized search

## Abstract

Recommendations are increasingly used to support and enable discovery, browsing, and exploration of items. This is especially true for entertainment platforms such as Netflix or YouTube, where frequently, no clear categorization of items exists. Yet, the suitability of a recommendation algorithm to support these use cases cannot be comprehensively evaluated by any recommendation evaluation measures proposed so far. In this paper, we propose a method to expand the repertoire of existing recommendation evaluation techniques with a method to evaluate the discoverability and navigability of recommendation algorithms. The proposed method tackles this by means of first evaluating the discoverability of recommendation algorithms by investigating structural properties of the resulting recommender systems in terms of bow tie structure, and path lengths. Second, the method evaluates navigability by simulating three different models of information seeking scenarios and measuring the success rates. We show the feasibility of our method by applying it to four non-personalized recommendation algorithms on three data sets and also illustrate its applicability to personalized algorithms. Our work expands the arsenal of evaluation techniques for recommendation algorithms, extends from a one-click-based evaluation towards multi-click analysis, and presents a general, comprehensive method to evaluating navigability of arbitrary recommendation algorithms.

## Background

Websites with large collections of items need to support three ways of information retrieval: (1) retrieval of familiar items; (2) retrieval of items that cannot be explicitly described, but will be recognized once retrieved; and (3) serendipitous discovery [1]. For a website with a large collection of items, such as an e-commerce website or a video platform, (1) can be enabled with a full-text search function. For (2) and (3), however, a search function is generally not sufficient. These types of information retrieval are, therefore, often supported by recommendations that connect items and enable discovery and navigation.

Users have been found to enjoy perusing item collections such as e-commerce sites or recommender systems without the immediate intention of making a purchase [2]. Flickr users predominately discover new images via *social browsing* [3]. More generally, some users prefer navigation to direct search even when they know the target [4]. In exploratory scenarios, the knowledge gained along the way provides context and aids in learning and decision-making [5, 6]. For platforms, where users immediately consume content, such as YouTube or Quora, recommendations serve the use case of *unarticulated want*, and are, therefore, a crucial part of user experience [7]. Moreover, for a range of entertainment platforms such as YouTube or Netflix, no clear structuring of items exists, and recommendations play a vital role in the user interfaces. If videos do not have any metadata or tags, navigation can be the only possible way of finding them. It is, therefore, critical for these systems to support discovery via links.

**Fig. 1:**
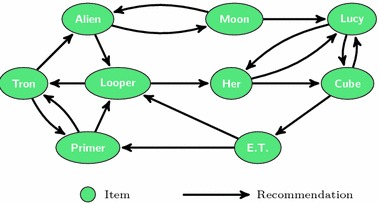
Recommendation network. When a website associates recommendations with each item, it forms a *recommendation network*—an implicit view of a recommender system, where nodes are items and edges are directed recommendations. Many websites associate a fixed number of recommendations with each item, which leads to a constant outdegree for each node in the network. The illustration shows a scenario, where two recommendations are available for each movie

The links generated by a recommender system are, by their very conception, meant to be navigated and used for exploration and navigation. When a website provides recommendations along with each item, the items and the associated recommendations form a *recommendation network*—an implicit view of a recommender system, where items are nodes and recommendations are edges. Figure 1 shows an example of such a network. This type of recommendations is frequent on e-commerce websites, such as Amazon’s “customers who bought this also bought”.

Knowing more about recommendation networks would give website operators the possibility to assess the effects of recommendations and help to produce recommendations that make it easier for users to discover and explore items. While a few studies have already looked at recommendation networks and provided first important insights into the nature and structure of these networks [8–11], there is no systematic approach to evaluating the network effects of recommendation algorithms both statically (discoverability) and dynamically (navigability).

The main contribution of this paper is a general method for evaluating navigability of arbitrary recommendation networks via both topological analysis and the evaluation of navigation models by simulation. The application of established techniques from network science allows us to present a novel method that extends common evaluation measures towards a path-based evaluation and expands the arsenal of existing recommendation evaluation techniques with two dimensions that have not received sufficient attention so far.

The method consists of two parts: first, we analyze *discoverablity*, the property of a recommendation algorithm to enable users to reach items. We evaluate it by looking at aspects of the recommendation network topology, namely, components, bow tie structure and path lengths.

Second, we investigate *navigability*, which measures the degree to which a recommendation algorithm is able to assist users to actually navigate and explore an item collection. We evaluate the practical navigability of a recommendation network using simulations based on three navigation models established in the literature, namely, *point-to-point navigation* [12], *navigation via berrypicking* [13], and *navigation via information foraging* [14].

This method is an extension of an evaluation method for navigability of recommendation algorithms of the previous work by the authors [15].

We show the feasibility of this method by applying it to four non-personalized recommendation algorithms on three data sets and investigate their properties. However, our method is not limited to evaluating non-personalized recommendation algorithms, but can be applied to any recommendation algorithms including personalized instances. We, therefore, illustrate the general suitability of our method and report initial results on personalized recommendations.

## Related work

Related work to this paper can be grouped into three parts: evaluation of recommeder systems, network science, and network-theoretic evaluation of recommender systems.

### Evaluation of recommender systems

Initially, recommender systems were mostly evaluated in terms of prediction accuracy [16]. However, the focus on accuracy has been found to neglect other import applications of recommender systems such as support for the discovery of novel items, browsing, or learning about diverse recommendations from related genres, and may lead to a bias towards popular items [9, 17] or a filter bubble effect [18]. For these reasons, a vast array of evaluation metrics for additional properties of recommender systems has been developed.

#### Prediction accuracy

The prediction accuracy is measured by comparing ratings predicted by the recommendation algorithm to a withheld set of actual user ratings and computing the deviation, for example, with the root-mean squared error (RMSE). Accuracy metrics have traditionally received the most attention in the evaluation of recommender systems [16].

#### Diversity

A recommendation list consisting only of very similar (e.g., all Star Trek films) can have a high prediction accuracy, but actually a low utility for users. Diversity measures the difference among a set of jointly shown recommendations and can be regarded as the opposite of similarity [19, 20]. Diversified recommendations have been found to lead to increased user satisfaction [21].

#### Novelty

Much like a lack of diversity, recommending only well-known (popular) items to users is of little use. Metrics for novelty refer to the difference between past and present experiences [16, 20, 22] and measure the degree of recommendations leading to unfamiliar items.

#### Serendipity

Serendipity, or pleasant surprise, measures the fraction of recommendations that are both novel (surprising) and relevant (interesting) [2, 16, 23].

#### Coverage

Coverage describes how many items a system can generate recommendations for (*prediction coverage*), and how many items are effectively ever recommended to users (*catalog coverage*) [2, 16, 23]. As such, coverage is a simple measures that shows how many items a recommendation algorithm renders discoverable.

### Network science

To evaluate discoverability and navigability, we make use of approaches from network science. Ever since Milgram’s small-world experiments [24], researchers have been making efforts to understand *navigability* and in particular *efficient navigation* in networks. Kleinberg [12, 25] and Watts [26] formalized the property that a navigable network requires short paths between all (or almost all) nodes. Formally, such a network has a low diameter bounded by a polynomial in log(*n*), where *n* is the number of nodes in the network, and a giant component containing almost all the nodes exists. In other words, because the majority of network nodes are connected, it is possible to reach all or almost all of the nodes, given global knowledge of the network. The low diameter and the existence of a giant component constitute necessary topological conditions for network navigability. In this paper, we apply a set of standard network-theoretic measures to assess if a network satisfies them.

Kleinberg also found that an *efficiently navigable* network possesses certain structural properties that make it possible to design efficient local search algorithms (i.e., algorithms that only have local knowledge of the network) [12]. The delivery time (the expected number of steps to reach an arbitrary target node) of such algorithms is then sub-linear in *n*. In this paper, we investigate the efficient navigability of networks through the simulation of a range of search and navigation models.

### Network-theoretic evaluation of recommender systems

The static topology of recommendation networks has been extensively studied for the case of music recommenders. Their corresponding recommendation networks have been found to exhibit heavy-tail degree distributions and small-world properties [8], implying that they are efficiently navigable with local search algorithms. Celma and Herrera [9] found that collaborative filtering provided the most accurate recommendations, while at the same time made it harder for users to navigate to items in the long tail. A hybrid recommendation approach and content-based methods were able to provide better novel recommendations. These results suggest that a trade-off exists between accuracy and other evaluation metrics. For movie recommendations, Mirza et al. [27] proposed to measure discoverability in the bipartite recommendation graph of users and items as an evaluation measure.

A first study [10] has already explored the discoverability and reachability of the recommender systems of IMDb using an analysis method similar to the one presented in this work. The corresponding recommendation networks were shown to generally lack support for navigation scenarios. However, the use of diversified recommendations was able to substantially improve this and lead to more navigable recommendation networks. While these analyses have shown certain topological properties and first aspects of navigability, we still know very little about the dynamics of actually using recommendations to find navigational paths through a recommender system.

## Methods

In the following, we describe the general approach, the data sets, recommendation algorithms we use and how we derive the corresponding recommendation networks.

### General approach

In this paper, we propose a method that first assesses *discoverability* and then *navigability* of a recommendation algorithm:

1. *Discoverability* Discoverability is the property of a recommendation algorithm to enable users to reach items. To evaluate it, we examine the static topology of a recommendation network and evaluate the discoverability by means of the bow tie structure and path lengths.
2. *Navigability* Navigability measures the degree to which a recommendation algorithm is able to assist users to actually navigate and explore an item collection. We evaluate the practical navigability of recommendation networks using simulations based on three different navigation models established in the literature: (a) *point-to-point navigation* [12] as an example of goal-oriented navigation with a single fixed goal; (b) *navigation via berrypicking* [13] as an example of goal-oriented navigation with multiple and variable goals; and (c) *navigation via information foraging* [14] as an example of exploration.

The code for this method is open source and available on GitHub.^1^

### Data sets

We use two types of items (namely, books and movies) from three data sets for this paper.

*MovieLens* 2 is a film recommender system maintained by GroupLens Research at the University of Minnesota. For this work, we use the data set consisting of one million ratings^3^ from 6000 users on 4000 movies. Each user in the data set has rated at least 20 movies.

*BookCrossing* is a book exchange platform.^4^ For this work, we use a 2005 crawl of the website [21]. As a preprocessing step, we filter out implicit ratings and combine the ratings of duplicate books with identical titles and authors. Furthermore, to be able to obtain meaningful results from the recommendation algorithms, we condense the data set and only keep ratings from users who rated at least five books and books which were at least rated 20 times. This leaves us with roughly 50,000 ratings by 1088 users on 3637 books.

*IMDb* is a database about movies and TV shows.^5^ We use a 2015 crawl of the website [10], from which we use all items published in the years of 2013 and 2014. We again condense the data set and only keep ratings from users who rated at least five books and books which were at least rated 20 times. This yields a data set of 2,254,873 ratings for 6690 titles by 37,216 users.

### Recommendation algorithms

We calculate recommendations in the following way: for a given set of items *I* and a recommendation algorithm *R*, we use *R* to compute the pairwise similarities for all pairs of items $(i, j) \in I$. For each item $i \in I$, we then define the set of the top-N most similar items to *i* as $L_{i, N}$. We investigated $N \in \left[ 1, 20\right]$, which we consider a plausible range for recommender systems. We then create a directed top-N recommendation network $G\left( V, N, E\right)$, where $V = I$, *N* is the number of recommendations available for each item and $E = \{ \left( i, j\right) | i \in I, j \in L_{i, N}\}$. This method leads to recommendation networks with constant outdegree and varying indegree—representing a typical setting for top-N recommendations such as Amazon.com’s *Customers Who Bought This Item Also Bought*.

For simplicity’s sake, we investigate recommendation algorithms based on non-personalized recommendations. The similarities these recommendations are based on, however, are directly taken from the similarities used in the recommendation algorithms. They, therefore, represent the recommendations (and the recommendation networks) as an unregistered or newly registered user would see them. For most websites, the vast majority of visitors does not contribute or register—this is known as the *participation inequality* or the *90-9-1 Rule* (90% lurkers, 9% intermittent contributers, and 1% heavy contributers) [28–30]. It seems likely that, for example, YouTube only has little preference information from about 90% of its visitors and, therefore, frequently needs to show non-personalized recommendations. However, our method is general and also applicable to personalized recommendation algorithms. We exemplarily demonstrate this in section  and report first results.

We use each of the following four recommendation algorithms in this work.

#### Association rules (AR)

Association rules are based on the market-basket model, where, in this case, we put all items rated by the same user into a basket and regard ratings as binary only (i.e., rated/not rated). For every ordered pair of items (*i*, *j*), we then evaluate a simple algorithm inspired by the Apriori algorithm [31] and rank all items by how much more likely an item is to be consumed if another item was consumed. Specifically, we compute the fraction of co-ratings of *i* and *j* over the total ratings of *i* (i.e., the fraction users who rated both *i* and *j*, out of those who rated *i*). Let $U_i$ be the set of users who rated item *i*. We can then compute this as as $\frac{|U_i \cap U_j|}{|U_i|}$. This is also known as the *confidence* of an association rule. To compensate for the popularity of *j*, we then divide by the fraction of users who did not rate *i* but still rated *j*. Let $\overline{U}_i$ be the set of users who did not rate item *i*. We can then divide by $\frac{|\overline{U}_i \cap U_j|}{|\overline{U}_i|}$ to counter the effect of highly popular items that are likely to be co-rated with every item, but would not be very useful as a recommendation. We then take the top-*N* items most likely to be co-rated with an item by this measure.

#### Collaborative fltering (CF)

Collaborative filtering is a recommendation algorithm that predicts ratings based on similar users or items. The variant we used is inspired by item-based collaborative filtering, where for a given user $u \in U$, the prediction for a rating for an unrated item $i \in I$ is computed based on a small number of *k* items $j_1, \dots j_k$ that *u* has already rated. These similar items are selected as *k* most similar ones to *i*, based on the similarity of their rating vectors. A rating vector for item *i* has |*U*| dimensions, one for every user, and its elements are zero except for the ratings assigned by the users to the item *i* (where the ratings are integers between 1 and 5). The predicted rating for *i* by *u* is then computed as the sum of the ratings of $j_1, \dots j_k$ weighted by their similarity to *i*. A common similarity measure used to this end is the centered cosine similarity, which for two vectors *x* and *y* is defined as

1 $$\begin{aligned} \text {CenteredCosSim}(x, y) = \frac{(x - \bar{x}) \cdot (y - \bar{y})}{||x - \bar{x}|| ||y - \bar{y}||}, \end{aligned}$$

where $\bar{x}$ stands for the vector mean of *x*. To obtain non-personalized recommendations, we compute the centered cosine similarity between an item *i* to all other items and recommend the top-*N* items $j_1 \dots j_N$.

#### Interpolation weights (IW)

Interpolation weights are applied to predict ratings in the same way as item-based collaborative filtering [32]. A prediction for an item *i* and a user *u* is computed based on a small number of *k* items $j_1, \dots j_k$ that *u* has already rated. These *k* items are selected to be the most similar ones to *i*. However, instead of using a predefined similarity measure for this task (such as the centered cosine similarity), the similarity function that represents the similarity between pairs of items is learned from the data. The rating values of the top *k* most similar items are then weighted by this similarity function and added up to yield the predicted rating. The similarity function between pairs of items is represented by a matrix *W* of dimensions $|I| \times |I|$, the elements of which are also known as *interpolation weights*. A predicted rating $\hat{r}_{u, i}$ is then computed as

2 $$\begin{aligned} \hat{r}_{u, i} = \sum _{j \in N (u, i)} w_{i, j} r_{u, j}, \end{aligned}$$

where *N*(*u*, *i*) is the *neighborhood* of item *i* (i.e., the *k* most similar items). The matrix *W* is initialized uniformly at random. The set of known ratings by users for items is then split into a training set (containing 80% of the ratings) and a test set (containing the remaining 20%). The weights in the matrix *W* are then iteratively adjusted by gradient descent, in terms of the ratings from the trainings set. More specifically, the gradient is computed based on the root-mean square error (RMSE) between the predictions and the ratings. The algorithm is then evaluated by comparing the predictions for the test set. For the non-personalized recommendations, we take the learned interpolation weights as the similarity measure to identify the *N* most similar items for each item.

#### Matrix factorization (MF)

Matrix factorization is a latent factor model and describes both items and users of a recommender system by relations to a small number of latent factors [33]. To this end, the rating matrix *R* (of dimensions $|U| \times |I|$) is approximated as $R = Q P^T$, where *Q* and *P* represent the relations between items and users with the latent factors. By learning the factors for a training set of known items, the full matrix *R* (i.e., all possible ratings) can be approximated. *Q* is of dimensions $|I| \times f$ and $P^T$ is of dimensions $f \times |U|$, where *f* is the number of latent factors. A prediction for the rating $\hat{r}_{u, i}$ is computed as

3 $$\begin{aligned} \hat{r}_{u, i} = q_i \cdot p_u, \end{aligned}$$

where $q_i$ is a row vector from *Q* that contains the relations between item *i* and all latent factors, and $p_i$ is a column vector from $P^T$ that contains the relations between the user *u* and the latent factors. To learn *Q* and $P^T$, we initialize them uniformly at random. Like for the computation of the interpolation weights, the given ratings are then split into a training and test set. The matrix *R* is then approximated as $Q P^T$, and these approximations are iteratively improved by gradient descent, based on the RSME between the predicted and the actual ratings. After the learning step, we compare each item with every other item to find the most similar ones. To this end, we use the vector of latent factors as the description for an item and compute the centered cosine similarity with all other items this way. We then take the top-N most similar items to obtain non-personalized recommendations.

## Evaluating discoverability

The first step of our proposed evaluation method assesses the discoverability of a recommendation algorithm, which measures the static reachability of items in a recommender system and represents a prerequisite for efficient navigability. We evaluate discoverability in two parts: *effective discoverability* (bow tie structure) and *efficient discoverability* (path lengths).

### Effective discoverability

**Fig. 2:**
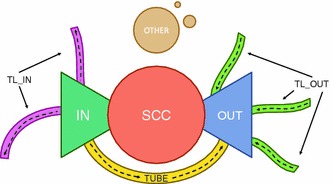
Bow tie model. The bow tie model [34] partitions a network into a strongly connected component (*SCC*), flanked by *IN*, where nodes can reach the core but are not reachable from it and *OUT*, where nodes are reachable from the core but not vice versa. Further components are *TUBE*, providing an alternative route from *IN* to *OUT* and the *TENDRILS (TL_IN, TL_OUT)* which contain nodes connected to *IN* and *OUT* which cannot reach the *SCC*. Any remaining nodes are collected in *OTHER*. The colors in this figure correspond to Fig. 3

**Fig. 3:**
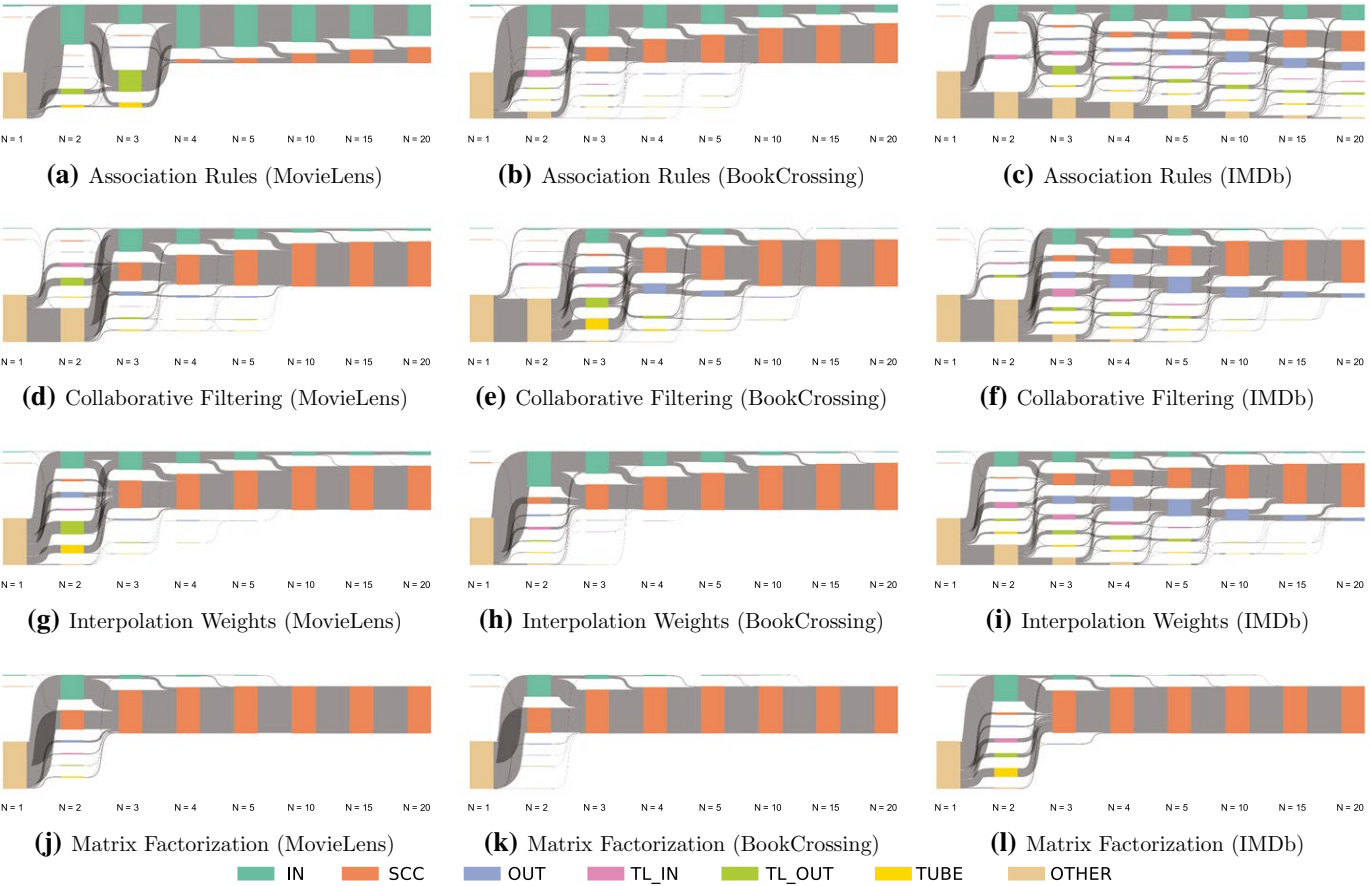
Bow tie membership over *N*. The figures depict the memberships of items to the components of the bow tie model (see Fig. 2), and the changes to membership as the number of recommendations *N* at each item increases from one through twenty. Each component is of the size proportional to the number of items it contains, with all components adding up to $100\%$. Except for the association rules recommendations, most nodes belong to the *IN* or *SCC* component. This is beneficial for discoverability, as this means there exists paths between most pairs of items (in case of *SCC*), or that, at least, almost any item in the system allows reaching the strongly connected set of items

#### Description

The analysis of the partition with the bow tie model allows us to assess the *effective discoverability* of a recommendation algorithm. This model is a prominent model for the partitioning of a directed network, originally developed for the analysis of the Web [34]. The model partitions a network into three major components: the largest strongly connected component (*SCC*), wherein all nodes are mutually reachable, a component of all nodes from which *SCC* can be reached (*IN*) and a component of all nodes reachable from *SCC* (*OUT*). Figure 2 shows the model in more details and explains the components. Note that the components of the bow tie model do not necessarily correspond to components in a network-theoretic sense: while the *SCC* does form a strongly connected component, for example, the *IN* component generally consists of multiple components. This implies that the *SCC* is reachable from any node in *IN*, but not all nodes within *IN* are mutually reachable. The *IN* component of the bow tie model, therefore, represents one-way navigational flows in the network.

#### Results and interpretation

Figure 3 shows the bow tie membership over *N* (i.e., the number of recommendations available at each item). In general, the size of the *SCC* (i.e., the largest strongly connected component) in the recommendation networks grows with *N*. This follows from the increasing density in the network—in fact, as *N* increases, at some point, all items are bound to end up in the *SCC*. The size of the *SCC* is related to *catalog coverage* [23], which measures the fraction of items which are recommended. However, it also measures the size of the largest set of items that are not only recommended but also mutually reachable and, therefore, discoverable.

In real-world examples, the number of immediately visible recommendations typically lies between 4 and 12. For instance, Amazon recommends between five and eight items (depending on screen resolution), YouTube recommends 12 videos and IMDb lists six related films. If our examples generalize to these data sets, this comparison shows that standard recommendation approaches with five recommendations at each item allow users to explore between 11 and 99% of all items (cf. Fig. 3). For 20 recommendations, the sizes of the *SCC*s increase to 43–100%. Discoverability, therefore, depends on both the number of recommendations and the choice of algorithm.

The recommendations generated by association rules result in an *SCC* of $11\%$ (MovieLens), $59\%$ (BookCrossing) and $14\%$ (IMDb) for five recommendations. With 20 recommendations at each item, this percentage somewhat improves to 34, 84, and 43%. For the other algorithms at $N=5$ recommendations, the *SCC* sizes range from 75 to 99%, thus providing better effective discoverability in the resulting networks. For $N=20$, the sizes further increase. Overall, the recommendations generated by matrix factorization perform best and lead to close to $100\%$ of items in the *SCC* for all values of $N \ge 5$.

The recommendations for the IMDb data set lead to a visibly more fragmented bow tie structure of the networks. A potential explanation for this lies in the sparsity of the data set: the rating matrix for IMDb contained just $0.91\%$ of all possible entries, whereas for the other data sets, this was the case for $4.16\%$ (MovieLens) and $1.26\%$ (BookCrossing). Furthermore, the larger number of users in the IMDb data set leads to a substantially smaller fraction of possible co-ratings between items being present, thus making it more difficult for the association rules, collaborative filtering, and interpolation weights algorithms, which rely on co-ratings to generate the recommendations. As a result, the recommendation networks also show a substantially larger clustering coefficient than the other data sets. This does not occur as strongly for the matrix factorization algorithm, as this algorithm learns associations between items and latent factors. Therefore, if two items were never co-rated by any user, but still share a strong association with common factors, they are still deemed similar and can be recommended. However, the recommendation networks for IMDb generated by matrix factorization do also show larger clustering coefficients than the other data sets, indicating the presence of a number of densely interconnected (clustered) regions.

Even when a larger number of recommendations is present, users tend to prefer the ones at the top of a list [35].

For this reason, we also look at the results for $1\ldots 4$ recommendations. The first thing that stands out is the stronger fragmentation of these networks. For just one recommendation, discovery of items in the networks is hardly possible, as one recommendation per item is not enough to form connected components. For two recommendations, discovery is at least partially enabled, in particular for matrix factorization, where for BookCrossing ($53\%$) and MovieLens ($40\%$), a substantial share of the items is already in a mutually reachable component. For all algorithms except association rules, four or five recommendations lead to fairly navigable networks for all investigated data sets. This suggests that when decluttering interfaces, a minimum of four or five recommendations should be kept to keep the system discoverable.

Apart from the *SCC*s, Fig. 3 shows that, overall, the dominant components are *IN* and *SCC*, except for fewer than five recommendations, where the networks are more fragmented. This implies that the network mainly consists of a core and items with recommendations leading to it. A detailed analysis of where links from *IN* component lead to underlines this intuition: In all networks for $N=5$, more than $68\%$ of all links from items in *IN* point to the *SCC*, and for $N=20$ , this is the case for more than $74\%$. From a navigational perspective, this means that items in the *SCC* can be directly reached from most items, but items in *IN* are in many cases only reachable by direct selection, e.g., via search results. We also find that for collaborative filtering and interpolation weights, the items in the *SCC* have a higher number of ratings than the ones in the remainder of the network. This could contribute to explaining a popularity bias identified in recommender systems [9, 17].

In addition, the *OUT* component include a relevant number of nodes for some combinations of algorithms and data sets. For the case of collaborative filtering and BookCrossing for $N = 5$, two separate strongly connected components with different sizes emerge: *SCC* and *OUT*. An explanation for this situation could again be found in the average number of ratings for items, which was substantially higher for items in the *SCC*. As collaborative filtering recommendations are calculated based on the centered cosine similarity, items with few co-ratings are more likely to reciprocate their recommendations for other items with only few ratings, and popular items with many co-ratings are more likely to recommend other popular items. This makes items in *OUT* more likely to remain in that component.

Likewise, for the IMDb networks, the items in the *OUT* component again were also rated less frequently than the ones in the *SCC*. To improve discoverability for collaborative filtering, the bow tie analysis could be used to introduce specific recommendations to better connect the network.

#### Findings

We find that the discoverability depends on both the number of recommendations shown (the more the better) and on the recommendation algorithm, where matrix factorization perform best. In terms of the bow tie structure, we find that the networks are dominated by a strongly connected core of items together with an *IN* component leading to it. This implies that items in the core are reachable from most items. Constructing navigable recommender systems could potentially be facilitated with the help of a modified algorithm to specifically recommend items based on this analysis.

### Efficient discoverability

#### Description

As the second step in evaluating discoverability, we investigate how *efficiently* recommendation algorithms enable item discovery.

To obtain insight into distribution of the shortest paths, we examine the path lengths between nodes in the recommendation networks. For a node *i*, we compute its median distance to other nodes as

4 $$\begin{aligned}\text{d}_ {\text{median}}(i) = \underset{i, j \in \text {SCC}(G)}{\mathrm {median}} \text{d}(i, j) \end{aligned}$$

where d(*i*, *j*) is the geodesic distance between *i* and *j*. We investigate the distribution of median path lengths for all items located in the *SCC* of recommendation networks. This provides us with a means of assessing the efficiency of discoverability from the path lengths.

**Fig. 4:**
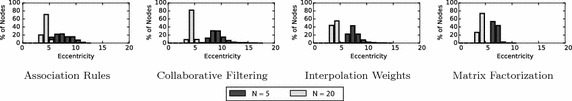
Median distance distributions. The median distance of a node measures the median shortest path from that node to any other node in the largest strongly connected component and allows us to gain insights into the lengths of common paths in a recommendation network. This figure shows the median path lengths for MovieLens (the other data sets lead to very similar results). As the number of recommendations increases from 5 to 20, path lengths substantially decrease. This shows that the number of recommendations exerts a strong influence on the resulting path lengths

#### Results and interpretation

Figure 4 plots the distribution of the median path lengths of all nodes in the largest components for $N = 5$ and $N = 20$ recommendations for MovieLens. The other data sets are qualitatively very similar. Overall, we find that increasing the number of recommendations leads to smaller distances in the recommendation networks. This confirms that the number of recommendations shown has a substantial influence on discoverability.

For all recommendation networks we investigate, the sizes of the largest strongly connected component (within which the path lengths were computed) increase as *N* is raised from 5 to 20. For example, for the recommendations for BookCrossing generated by association rules, the size of the largest strongly connected component increases from 59 to 80% of all items. Despite this, the median path length decreases from 7 to 4. However, this phenomenon has actually been observed for many types of graphs [36]. A possible explanation can be found in the increasing density of the networks: even though the largest strongly connected component increases in size, the number of recommendations for each item also strongly increases. This enables additional paths between items.

The diameters (the maximum path lengths in the *SCC*s) they range from 12 to 38 for $N=5$ and 7 to 25 for $N=20$. Large distances between pairs of nodes in a recommendation network such as these raise the question of whether users would actually undergo click sequences of this length to navigate the items. Analysis of Wiki game data, where players actively try to find shortest paths, has shown that humans need an average of three clicks more than the shortest possible paths [37]. To compare, the maximum of medians range from 7 to 28 for $N = 5$ and 4 to 17 for $N = 20$.

In terms of recommendation algorithm, matrix factorization leads to the shortest paths, followed by interpolation weights. To investigate the influence of path lengths further, we now turn our attention to the evaluation of navigability and its practical aspects.

#### Findings

We examine the distributions of shortest paths between nodes in the largest components and find that the number of recommendations exerts a strong influence on the resulting path lengths. Some of the distances between nodes (up to 38 hops) are potentially too long for reasonably efficient navigation. Matrix factorization and interpolation weights lead to the shortest distances.

## Evaluating navigability

As the second step of our analysis, we now focus our attention on the navigation dynamics of recommendation algorithms.

A defining property of online navigation is that the knowledge users have about a website is mostly local: users only perceive the links emanating from the current page and generally only have intuitions about where those links might lead, but lack global knowledge about the system. In the case of a top-*N* recommender system, users are generally only aware of the recommendations provided with the current item.

In a typical information seeking model, users move from one item to another by following links. This activity can be intertwined with using the search function—e.g., exploring the results, backtracking and trying another path or simply entering a refined search query in the search field [38]. In what follows, we evaluate simulations of navigation in recommender systems and measure the navigational success rates. This evaluation goes beyond a standard one-click evaluation scenario in recommender systems—it is in particular an inspection of the suitability of these networks to accommodate users in following several sequential recommendations, one after the other.

### Simulation methods

To model navigation, we apply a *greedy search* approach. This search algorithm that takes its name from its action selection mechanisms. At each step, the algorithm evaluates a heuristic for every present link and greedily selects the one maximizing that heuristic. The implementation for the simulation that we used was also capable of marking visited items and only visits each item once. In case no unvisited item is present, the simulation backtracks to the previously visited item. Greedy search has been used in the previous work to analyze navigation dynamics in networks [39, 40] and found to produce comparable results to human navigation patterns [41, 42].

The heuristic we use is the TF-IDF cosine similarity. TF-IDF for a document *d* (which for our case corresponds to a textual description for an item *i*) and a term *t* is defined as

5 $$\begin{aligned} \text {TF-IDF}(t, d) = \text {TF}(t, d) \cdot \text {IDF}(t), \end{aligned}$$

where the *term frequency* $\text {TF}(t, d)$ is the number of occurrences of term *t* in document *d*, and the *inverse document frequency* $\text {IDF}(t)$ of a term *t* models the inverse of the number of documents a term occurs in, and is defined as

6 $$\begin{aligned} \text {IDF}(t) = \log \frac{1 + D}{1 + \text {DF}(t)} + 1, \end{aligned}$$

where *D* is the number of documents and the document frequency $\text {DF}(t)$ is the number of documents a term occurs in. This definition follows the one from *scikit-learn*, the Python library that was used for this paper.^6^ To compare the text of documents, each document is represented by a vector with elements corresponding to the 50,000 most frequent terms across all documents. These vectors are then compared with the cosine similarity, which for two vectors *x* and *y* is defined as

7 $$\begin{aligned} \text {CosSim}(x, y) = \frac{x \cdot y}{||x||\,||y ||}. \end{aligned}$$

This has the advantage of assigning a higher weight to terms that are descriptive of a document and of attenuating the influence of common stopwords.

For this paper, we used the item title plus a brief textual description to compute the TF-IDF cosine similarity. At each step, the simulation selects the link leading to the item that has the highest TF-IDF cosine similarity to the navigation goal. As the text for BookCrossing, we used the summary provided for each book at GoodReads,^7^ a social cataloging site for books, and for MovieLens and IMDb, we used the title, brief plot summary, and the storyline description present for the movies at IMDb. We take these similarities to represent vague intuitions about navigation that users might gain from looking at the titles and descriptions of recommendation targets. For example, if a user was looking for a new science-fiction movie, they might be tempted to follow recommendations to other science-fiction movies based on the title, a brief corresponding textual description or the displayed image. We use intuitions based on a measure that we assume to be independent of ratings to decouple the intuitions from the ratings and to be able to fairly evaluate all algorithms.

Greedy search is deterministic, as it always greedily selects the best next node, but there exists a variety of stochastic variations [39]. In addition to the deterministic approach, we also evaluated all simulations with an $\epsilon$-greedy approach, in which the next node is selected uniformly at random with a random chance of $5\%$, thus modeling a degree of uncertainty. However, this only led to minor changes in the results, and for sake of brevity, we only report the results for deterministic greedy search.

We evaluate a simulation for a total of 50 selection steps per navigation goal. When evaluating a specific website, this parameter should be tuned to the amount of clicks users can be expected to remain on the website (e.g., fewer for e-commerce sites and more for entertainment sites). The 50 steps we used stand in for users willing to dedicate some time to a website. For comparison, we also evaluated all simulations for 10 and 25 steps and found that, while the absolute success rates decreased, the relative differences between the approaches did not change. For sake of brevity, we only report the results for 50 steps.

We also evaluate two baseline solutions: an *optimal solution* makes use of the shortest possible paths in the network (that users with perfect knowledge of the network could take). A *random solution* performs a random walk with no background knowledge at all.

A number of information seeking models have been established in the literature. To investigate the general suitability of recommendation algorithms to navigation based on different approaches, we evaluate navigation scenarios based on three of these models:

- Point-to-point Navigation [12].
- Berrypicking [13].
- Information Foraging [14].

**Fig. 5:**
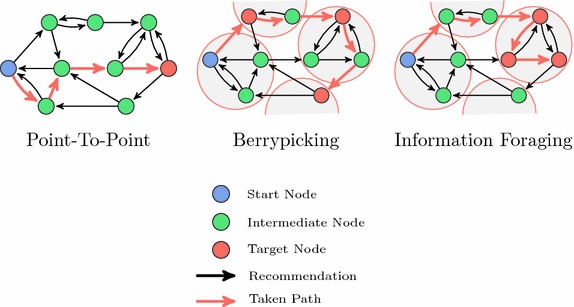
Information seeking scenarios. We use three information seeking scenarios to study navigability of recommendation networks. The objective in point-to-point navigation is to find a single goal item. For berrypicking, we cluster the networks and set the goal of finding any one item in four predetermined clusters (shown in gray). For information foraging, the goal is to find multiple items in a predetermined cluster. These scenarios cover the rediscovery and serendipitous discovery points of Toms’s ways of information retrieval [1]

Figure 5 shows examples of scenarios, which are explained in detail in what follows. For all scenarios, the start and target nodes in the network are determined independently of the network structure, i.e., regardless of whether the recommendation algorithm actually enabled a path between them. This allows us to fairly compare all recommendation algorithms and shows how well they support both discoverability and navigability. For sake of brevity, we report the results for five and twenty recommendations.

### Point-to-point navigation

#### Description

Point-to-point navigation represents the task of finding a single target item in a recommendation network and represents the navigational behavior of users with a specific item in mind that they cannot explicitly describe. For example, a user could try to find a science-fiction movie with a specific motif or to rediscover something on tip of their tongue. As such, this scenario covers point (2) (“retrieval of items that cannot be explicitly described but will be recognized once retrieved”) of Toms’s ways of information retrieval [1].

As start-target pairs we (a) randomly sample 1200 pairs of nodes from each network (random targets) and (b) sample 1200 pairs of nodes proportionally to how often they were rated together in the data sets (rating-based targets). We then evaluate navigation simulations for all of these pairs, starting at the start node of the pair and with the objective of reaching the target node.

#### Results and interpretation

**Fig. 6:**
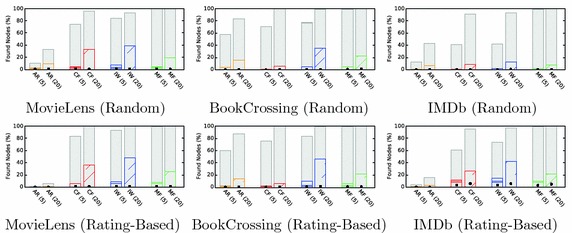
Success ratios for the point-to-point navigation scenario. The bars depict the average percentage of found target nodes for all start-target pairs in the scenarios. The first row depicts random start-target pairs and the second row pairs selected based on co-ratings. Baseline success rates are depicted as gray bars (optimal solutions) and black dots (random walk solutions). Recommendation networks generated by interpolation weights (IW) performed best

Figure 6 displays the success rate (i.e., the fraction of successful simulations). The first thing to note is the optimal solutions shown as gray bars. Since the number of steps per simulation (50) is larger than the distances between all start-target pairs in the recommendation networks, these correspond to all start-target pairs between which a path of any length existed. The optimal solution is, therefore, a measure of how well a recommendation algorithm theoretically supports this navigation scenario.

The second baseline approach is the random walk, which shows the success rates achievable by an uninformed random process and serves to demonstrate that the simulations based on greedy search are able to exploit the link selection heuristic to reach navigation goals. The simulations always achieve a better success rate than the random walk baseline.

Point-to-point navigation with greedy search for $N = 5$ recommendations leads to an average success rate of $3.95\%$ (random targets) and $5.92\%$ (rating-based targets). This indicates that users would be able to retrieve only a very small share of items in the recommender systems by focused point-to-point navigation. For $N = 20$ recommendations, the success rates increase substantially. Recommendations generated by interpolation weights lead to the best success rates, with 13–39% for random targets and 42–48% for rating-based targets. This again shows that the number of recommendations shown exerts a strong influence on the resulting navigability. The target selection also affects the success rates, with rating-based targets leading to substantially better outcomes. This follows from the fact that both the rating-based target selection and the recommendation algorithms made use of the co-ratings. The ranks of the recommendation algorithms, however, do not change: For both target sets, interpolation weights lead to the best results, followed by the recommendations generated by matrix factorization. For real-world recommenders, this shows that for the actually co-rated pairs of items, paths can be retrieved more easily.

#### Findings

We find that for five recommendations, the resulting recommendation networks are poorly navigable. Raising the number of recommendations increases the navigational success rates. For the data sets, we investigate recommendations by interpolation weights fare best.

### Navigation via berrypicking

#### Description

Berrypicking is an information seeking model proposed by Marcia J. Bates [13], which regards information seeking as a dynamic process.

In berrypicking, the information need is evolving and can be satisfied by multiple pieces of information in a *bit-at-a-time retrieval*—an analogy to picking berries on bushes, where berries are scattered and must be picked one by one. Berrypicking can be though of as covering points (2) (“retrieval of items that cannot be explicitly described but will be recognized once retrieved” and (3) (serendipitous discovery) of Toms’s ways of information retrieval [1]: the bit-at-a-time retrieval could aim at rediscovering a specific item or at serendipitously exploring items until an adequate item is found. Based on berrypicking, we evaluate a navigation scenario based on clusters, for which we study two approaches. For *genre-based clustering*, we aggregate based on decade of publication and genres. The publication date and genre information is supplied with the data set for MovieLens and IMDb, and for BookCrossing, we use the information from Goodreads, which allows its users to put books onto genre-based shelves, of which we use the top four. We then randomly sample subsets of four clusters for the berrypicking simulations. For *rating-based clustering*, we apply k-means based on the rating vectors for each item and select $k = |I| / 3$. We randomly pick a first cluster and randomly sample from one of the top four closest clusters based on Euclidian distance. We then repeat this based on the second and third clusters.

For both clustering approaches, we only use clusters consisting of 4–30 nodes and randomly choose one node from the first cluster as the starting point. The objective of the scenario is then to reach an arbitrary node from the second cluster, followed by an arbitrary node from the third and, finally, an arbitrary node from the forth cluster. In this way, the scenario models the evolving stages of berrypicking, where users inspect an item and adapt their information needs based on it.

As a difference to the point-to-point navigation scenario, the target of the navigation for the berrypicking scenario is not represented by a single node but by the centroid of the target cluster. The TF-IDF cosine similarity of a potential link target *l* is, therefore, represented by the average of the similarity between *l* and all items in the target cluster, i.e., intuitions about a group of items.

#### Results and interpretation

**Fig. 7:**
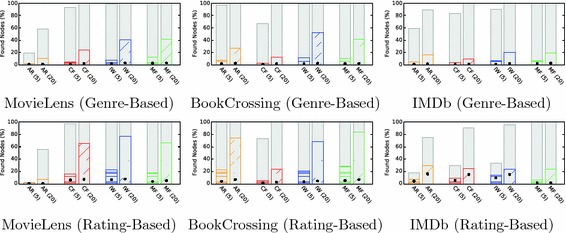
Success ratios for the berrypicking scenario. The bars depict the average percentage of found targets (genre-based clusters in the first row and rating-based clusters in the second row). Every simulation consisted of three target clusters, and the success rates are computed as the average number of found targets. Recommendation networks generated by interpolation weights (IW) and matrix factorization (MF) performed best

Similar to point-to-point navigation, for a small number of recommendations, none of the recommendation algorithms performs well (cf. Fig. 7). For five recommendations, the success rates for the case of genre-based clusters are comparatively low and range up to $12\%$. With 20 recommendations, this increases to up to $52\%$. Since the targets consists of three clusters, a success rate of $33\%$ indicates that, on average, one cluster was found and $66\%$ indicate an average of two clusters. The results, therefore, show that for 20 recommendations, nodes from one or two clusters are found. For the rating-based clusters, success rates are higher and range up to $83\%$.

The success rates for the IMDb data set are substantially lower than for the other two data sets. As the analysis for the effective discoverability has shown, the networks for IMDb are clustered more strongly than those of the other two data sets. For a dynamic information seeking scenario such as berrypicking, this means that the simulation of adapting information needs was not very well supported for IMDb. Like for the point-to-point navigation, interpolation weights lead to the best results overall. However, for a few cases, it is outperformed by matrix factorization. The reason for this likely lies in the better discoverability in the network generated by matrix factorization, which facilitates retrieving nodes from multiple clusters. With an average success rate of $23.75\%$, berrypicking was better supported than point-to-point navigation ($13.22\%$).

#### Findings

We find that the support for berrypicking, a scenario representing dynamic information search, is also not extensive for five recommendations, but improves for 20 recommendations. For rating-based cluster target selection, success rates range up to $83\%$, indicating a good support for evolving information needs. Interpolation weights and matrix factorization lead to the best results.

### Navigation via information foraging

#### Description

Information foraging [14] is an information seeking theory inspired by optimal foraging theory in nature, where organisms have adopted strategies maximizing energy intake. For instance, when foraging on a patch of food (e.g., apples on a tree), an animal must decide when to move on to the next patch (e.g., if reaching apples on the tree has become too strenuous). Some of the same mechanisms have identified for human information seeking behavior, as humans try to maximize the information gain. Information can be modeled as occurring in *patches*, and information seekers as guided by *information scent* [43]. Links leading to relevant targets are thought to emanate a stronger information scent than irrelevant links.

In a scenario based on information foraging, we model the scenario of depleting a patch of information. We assume that arriving at one of the nodes in an information patch, the objective is now to find other nodes in the patch—guided by information scent in terms of the TF-IDF cosine similarity. We take information foraging to model points (2) and (3) (“retrieval of items that cannot be explicitly described but will be recognized once retrieved” and “serendipitous discovery”) of Toms’s ways of information retrieval [1]. The implementation of the clustering and the TF-IDF cosine similarity to the targets was the same as for the berrypicking scenario.

#### Results and interpretation

A priori, it is not clear if retrieving multiple items from the same cluster represents an easier task than retrieving them from different clusters. A cluster of items does not necessarily mean that items are located in proximity in the recommendation network. However, the resulting success rates show that items from the same clusters in the network are easier to retrieve, and this indicates that the recommendation algorithms are able to use the characteristics in the ratings to support both genre-based and rating-based clustering.

**Fig. 8:**
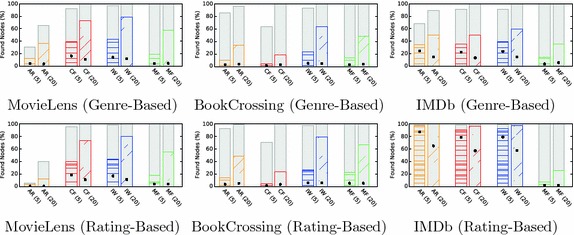
Success ratios for the information foraging scenario. The bars depict the average percentage of found targets (genre-based clusters in the first row and rating-based clusters in the second row). Every simulation consists of three target nodes, and the success rates are computed as the average number of found targets. Recommendation networks generated by interpolation weights (IW) performed best

Figure 8 shows that the success rates again measure the number of found items in a cluster. The results for this scenario show that the success rates for the baselines, namely, the random walks and the optimal solutions, are consistently very high. This also indicates that the network structures reflect the clustering very well. The results for the simulations ($44.01\%$ for the overall average) confirm this and lead to better results than is the case for the berrypicking scenario. Whereas for berrypicking, the simulations on the IMDb data set perform poorly, the contrary is the case for information foraging, where the success rates range up to $99\%$. This again confirms the strong clustering in these networks that lead to densely interconnected regions among similar items and that facilitated retrieval of items in the same cluster. This effect was stronger for the rating-based target clusters, which were created based on the same rating data than the recommendations. Recommendations generated by the interpolation weights algorithm generally fare best.

#### Findings

We find navigation via information foraging to be the best-supported scenario among the ones we investigated. With success rates up to $99\%$, retrieving items from the same cluster is very well supported. Interpolation weights lead to the best success rates.

## Personalized recommendations

In the previous sections, we have demonstrated the application of our proposed evaluation method to non-personalized recommendation algorithms. However, our method is not limited to them, but can be applied to any recommendation algorithm. To illustrate this, we now demonstrate the general suitability of our method to personalized recommendation approaches and report initial results.

### Description

The key difference for personalized recommendations is that a separate recommendation network emerges for every user based on the items that they have rated. To illustrate our method, we apply it to three types of users per data set: the users with the minimum, median, and maximum number of ratings. In addition, we need to decide how the personalized recommendations are selected among the possible recommendation candidates. For this illustration, we follow the approach of Amazon.com, as detailed by Linden et al. in 2003 [44]. The approach consists of two steps: first, a set of similar items is determined for each item. Second, the items with the highest predicted rating among this set are recommended. We study two examples of personalized recommendation approaches:

- *Pure* We compute a candidate set of similar items for an item—these are simply the non-personalized recommendations. Then, we select the *N* items from this set that have the highest predicted rating for the specific user.
- *Mixed* We again compute the set of similar items as for the pure recommendations, but only use the *N* / 2 recommendations with the highest predictions and the *N* / 2 top non-personalized recommendations (without introducing duplicates).

For both algorithms, we allow the recommendation of items that the user had already rated. This is another parameter that recommender system operators can tune. We note that if we do not allow this, the resulting recommendation networks show a decrease in navigability the more items a user has already rated. Allowing this, on the other hand, reduces the differences between the three user types, we investigated to minor differences. For sake of space, we, therefore, only report the results for the user type with the median number of ratings. We further only report results for a restricted set of parameters, namely, for the BookCrossing data set with recommendations generated by matrix factorization. The results for the other combinations of parameters were similar, but we leave it to future work to examine them in more details.

### Results and interpretation

**Fig. 9:**
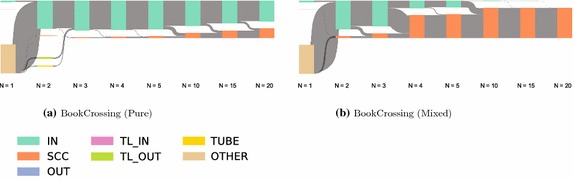
Bow tie membership over *N*. Using all personalized recommendations (pure) leads to lower discoverability than using an approach combining personalized and non-personalized recommendations. For personalization, the top $N+75$ recommendation candidates were computed for every item as candidates

**Fig. 10:**
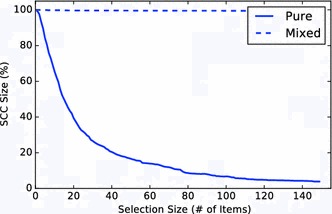
Dependence of SCC size on the number of recommendation candidates. To select personalized recommendations, we first compute the $N + S$ top non-personalized recommendations for each item. The recommendations are then selected as the ones that have the highest predicted rating for the user. *S* is a parameter we can choose—the higher *S*, the more responsive the recommendations become to a user’s preferences. However, increasing *S* leads to decreasing *SCC* size. This is caused by an increasing focus on preferred items for the user, leading to one-way structures in the networks. The same effect did not occur for mixed recommendations

The pure and mixed recommendation approaches lead to starkly different recommendation networks (see Fig. 9). Whereas the networks for the mixed algorithm resembled the ones for non-personalized recommendations, the pure algorithm leads to weakly connected networks with most of the items in the *IN* component. Furthermore, the size of the *SCC* for pure depended on the size of the set of recommendation candidates that were used to select the ones with the highest predicted ratings. The relationship was actually inversely proportional: the larger the set of candidates, the smaller the resulting *SCC* (cf. Fig. 10). This follows from the fact that the larger the set of candidates, the more often will the same items be chosen to be shown alongside multiple items, therefore, leading to a large *IN* component. This can be seen as a filter bubble effect. The mixed algorithm circumvents this problem by introducing non-personalized recommendations.

**Fig. 11:**
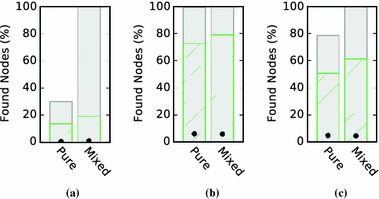
Navigational success rates. The figure shows the success rates for point-to-point navigation (**a**), berrypicking (**b**) and information foraging (**c**). All simulations were evaluated for BookCrossing, matrix factorization recommendations, 50 steps, and 20 recommendations at each item. The results show that while the mixed recommendations enable a better optimal solution, the recommendations did not reflect the intuitions of the navigation simulations very well

For the evaluation of navigability, Fig. 11 shows the evaluation for the rating-based targets and $N=20$ recommendations. The outcome is generally similar to non-personalized networks. The pure algorithm notably increases the success rates for the optimal solution, but not for the simulation results themselves. This indicates that while the mixed algorithm leads to a better discoverability in the networks, this was not necessarily the case for navigability. This in turn suggests that the recommendations generated by this algorithm did not capture the intuitions used in the navigation simulations very well. In future work, the method proposed in this paper could be used to develop a more effective personalized recommendation selections.

### Findings

We illustrate the applicability of our method to personalized recommendation algorithms by evaluating a set of sample parameters and data sets and find that for the parameters, we choose strong personalization actually leads to poorer discoverability and navigability.

## Discussion

We have presented a novel evaluation method that expands the repertoire of recommendation evaluation measures with a technique to evaluate discoverability and navigability. Our method is based on an evaluation conducted in two steps: the first step evaluates the discoverability by looking at the bow tie structure and path lengths. The second step evaluates the navigation dynamics of recommendation networks by simulating three different navigation models, namely, point-to-point navigation, navigation via berrypicking, and navigation via information foraging.

This method presents a comprehensive approach to evaluating the discovery and navigation dynamics in recommender systems. Particularly for websites such as Netflix or YouTube, where no clear ordering of items exists, recommendations play a vital part of the interface. For these websites, discoverability and navigability are critical aspects that cannot be properly captured by any of the previously proposed evaluation measures. Conducting the evaluation method proposed in this paper broadens our understanding of recommendation algorithms and leads to a more complete characterization of their properties.

To demonstrate the feasibility of our method, we applied it to three exemplary data sets and highlighted differences in discoverability and navigability for four different, non-personalized, recommendation algorithms. In general, we find that the number of recommendations available at each item has a substantial influence. For five recommendations, we find that the recommendation algorithms we investigate considerably limit the discoverability and navigability. With distances in the recommendation networks up to 38 hops, path lengths could be too long for users. In terms of navigation dynamics, our results show that five recommendations also severely restrict the retrieval of items. However, we also find that both properties can be improved by raising the number of recommendations. For the three navigation scenarios, we investigate we find that the explorative scenarios inspired by berrypicking and information foraging lead to the best retrieval performance, while the scenario based on point-to-point navigation was less well supported. While increasing the number of recommendations represents a simple solution, a large number of recommendations could potentially clutter the interface and overwhelm users [35]. This shows that there is still a substantial potential to improve recommendation algorithms to better support navigation dynamics.

As for the recommendation algorithms, we find that the recommendations generated by an interpolation weights and matrix factorization performed best overall. The association rule recommendations we investigated did not support discoverability and navigability very well and led to very fragmented recommendation networks. This suggests that exploiting the collective knowledge present in interaction of items and latent factors as done by interpolation weights and matrix factorization leads to more easily navigable recommender systems. However, more work is necessary to confirm these findings.

The recommendation algorithms selected for this work are established in the literature. Their selection was naturally arbitrary, but they serve the purpose of illustrating the evaluation and, therefore, do not limit our main contribution of presenting a novel evaluation method. We have shown the suitability of our method for non-personalized recommendation algorithms and thereby effectively inspected recommendation networks for users who are either new to the system or simply browsing without being registered. There is evidence that a large share of web users is not registered users and, therefore, only interacts with non-personalized recommendations. We also illustrated the applicability of our method for personalized recommendations by reporting the results of a sample combination of parameters and showed that perhaps, a bit counter-intuitively, increased personalization leads to less discoverable networks.

The navigation models applied in this method are well-established in the research community and cover a wide range of typical user interaction scenarios with information systems in general, and recommender systems in particular. Greedy search, the basis for our navigation scenarios based on these models, has been used in the previous work to analyze navigation dynamics in networks [39, 40] and has been found to produce comparable results to human navigation patterns [41, 42]. The navigation models we used do, however, have limitations and were deliberately kept simple, as the focus of our work was not on the information seeking models and their validity but on the properties of the recommendation algorithms. However, this does not limit our work, as our evaluation method does not depend on this particular model, which can easily be adapted or exchanged in future work. Possible enhancements to the navigation models could include a teleportation element modeling jumps between items without recommendations like in PageRank. This could be useful to represent the interplay with search function that also enables users to directly switch (jump) to arbitrary items. To model familiarization with a system, a learning component (e.g., for memorizing preferred paths) could be included. However, it should be noted that simplistic navigational models have been proven to be useful in many applications, such as PageRank.

In real-world information systems, recommendations are typically used in conjunction with other navigational links such as a navigational menu. Websites may also make use of other dynamically generated links such as *trending items* or *news items*. To study the navigational dynamics of sites of this type, it is necessary to look at the combination of all navigational aids. For example, it would easily be possible to add a navigational menu to the evaluations presented in this paper. This would likely have the effect of always leading to a fully connected network, as every page would then be connected to the home page. As this would also mask any navigational inefficiencies in the network, we believe that testing the recommendation algorithm on its own is still a useful addition to the toolkit for website operators.

## Conclusions

Our work extends common evaluation measures of recommendation algorithms towards a path-based evaluation. The presented method estimates the discoverability of items and assesses the navigability of the resulting recommendation network. Just as the evaluation of recommender systems has been shifting from accuracy-based measures towards diversification, coverage and time-dependent evaluations, we believe that our method helps push the frontier of recommendation algorithms towards producing recommendations that make it easier for users to discover and explore items.

While the results of our experiments are limited to the data sets, our method to evaluating the discoverability and navigability of recommendation networks is general. We have demonstrated our method extensively for non-personalized algorithms, but also shown its usefulness for personalized algorithms. It can be applied to arbitrary recommendation networks, thereby acting as a novel tool of measurement for an increasingly important dimension of recommendation systems.

## Footnotes

1
https://github.com/lamda/RecNet.

2
http://movielens.umn.edu.

3
http://grouplens.org/datasets/movielens/1m.

4
http://bookcrossing.com.

5
http://imdb.com.

6
http://scikit-learn.org/0.18/modules/feature_extraction.html#tfidf-term-weighting.

7
http://goodreads.com.
